# Isolated Intraocular Jarisch-Herxheimer Reaction Following Treatment for Syphilitic Chorioretinitis

**DOI:** 10.7759/cureus.67775

**Published:** 2024-08-25

**Authors:** Salil Mehta, Ameya Medhekar, Shambhavi Sarma

**Affiliations:** 1 Department of Ophthalmology, Lilavati Hospital, Mumbai, IND; 2 Department of Internal Medicine, Lilavati Hospital, Mumbai, IND

**Keywords:** cystoid macular edema, posterior uveitis, chorioretinitis, jarisch-herxheimer reaction, syphilis

## Abstract

A 39-year-old male patient presented with grossly reduced vision in the left eye for the past three months. Fundus evaluation revealed multiple discrete grayish-white deep chorioretinal lesions in the macular area. An optical coherence tomography (OCT) scan in the affected area was normal. Following investigations, a specific diagnosis of syphilitic macular chorioretinitis was made. A repeat OCT on day eight revealed cystic macular edema, consistent with a Jarisch-Herxheimer reaction (JHR). He was started on oral prednisolone in tapering doses and followed up till resolution. The ocular correlates of JHR are rare, and physicians should be aware of the potential of this phenomenon.

## Introduction

Syphilis is an increasingly prevalent infection that can cause a wide spectrum of ocular diseases, including intermediate uveitis, retinitis, and acute posterior placoid chorioretinitis [1]. The Jarisch-Herxheimer reaction (JHR) is an immunological reaction that commonly presents with symptoms such as fever, chills, or myalgias. Ocular correlates of JHR include optic disc or macular edema. We report the case of a patient who developed cystoid macular edema (CME) as a manifestation of JHR following treatment for syphilis.

## Case presentation

A 39-year-old male patient presented with grossly reduced vision in the left eye for the previous three months with intermittent oral ulcers. Significant medical history included renal calculi. His visual problems commenced three months earlier when he noticed a gradual reduction of vision in the left eye. He had sought an ophthalmologist’s opinion and was prescribed corticosteroid eye drops. Following several days of instillation, he was shifted to a month-long tapering course of oral prednisolone (40 mg daily). Still, he noticed no visual improvement and sought a second opinion at our center.

**Figure 1:**
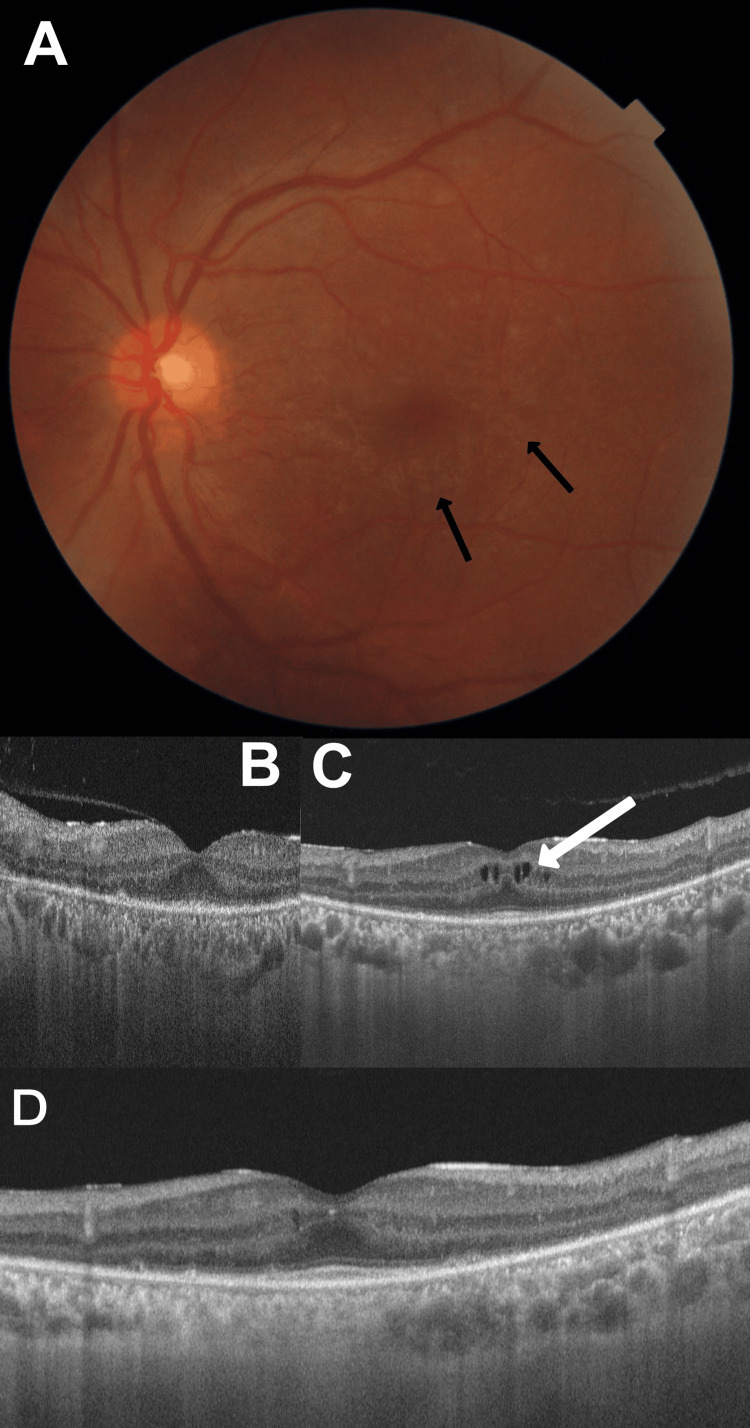
A) Color fundus photograph showing multifocal deep greyish-white chorioretinitis in the macula (black arrows); B) Initial OCT (day one) showing a normal macular appearance pre-treatment; C) OCT (6 mm radial scan) after eight days of penicillin treatment showing cystoid macular edema (white arrow); D) OCT (6 mm radial scan) at 42 days follow-up showing near-disappearance of the cystoid macular edema OCT: optical coherence tomography

On examination, his visual acuity was 6/6, N6 in the right eye, and counting fingers close to the face with accurate projection in the left eye. Slit-lamp examination was normal in either eye. Dilated fundus evaluation revealed normal findings in the right eye and multiple discrete greyish-white deep chorioretinal lesions in the macular area with a normal-appearing optic disc (Figure 1A). Perimetry revealed a normal visual field in the right eye and a complete loss of the visual field in the left eye. An optical coherence tomography (OCT) scan in the affected area was normal (Figure 1B). A provisional diagnosis of macular chorioretinitis was made, and intravenous methylprednisolone (1 gm daily for three days) was advised. Six days later, the visual acuity and fundus findings were unchanged. He was re-imaged, but no abnormalities were detected.

At this point, he underwent a panel of hematological and serological tests, which was notable for a positive Venereal Diseases Research Laboratory (VRDL) and rapid plasma reagin (RPR) test. On the advice of a physician, he was advised to undergo a confirmatory treponema pallidum hemagglutination (TPHA) test for syphilis, the results of which were positive. 

He was re-admitted for systemic antibiotic therapy. On admission, the examination of the genital, nervous, cardiovascular, respiratory, and cardiac systems was normal. A chest X-ray (posteroanterior (PA) view) was unremarkable. An MRI of the brain and orbits was normal. The patient's relevant laboratory results are presented in Table 1.

**Table 1:** The patient's laboratory parameters

Laboratory parameters	Test results	Reference values
Hemoglobin	14.50 gm/dL	13.0-17.0 gm/dL
White cell count	5500 cells/mm^3^	4-10,000 cells/mm^3^
Serum creatinine	1.09 mg/dL	07-1.4 mg/dL
C-reactive protein	20.87 mg/L	0-6 mg/L
Serum angiotensin-converting enzyme	22.2 U/L	12-68 U/L
Anti-nuclear antibody (ANA)	Positive (1:1000)	Negative
Venereal Diseases Research Laboratory (VDRL, serum)	Reactive (1:128)	Diagnostic titre >/= 1:8
Treponema pallidum hemagglutination (TPHA, serum)	1:5120	Negative
Mantoux test (10 purified protein derivative (PPD))	15x15 mm	Positive > 10 mm
Cerebrospinal fluid (CSF): microscopy	Normal	Normal
Tuberculosis-Mycobacteria Growth Indicator Tube (TB-MGIT)cultures	Negative	Negative
Aerobic cultures	Negative	Negative
Cytology	Few scattered small lymphocytes, and no malignant cells seen	No cells seen
Cryptococcus antigen	Negative	Negative
VDRL	Non-Reactive	Non-reactive
TPHA	Negative	Negative
Genexpert (*Mycobacterium tuberculosis* (Mtb)/rifampicin (RIF)	MTB complex not detected	MTB complex not detected
HIV duo	Negative	Negative
Random blood sugar	82.20 mg/dL	< 200 mg/dL
Viral serology; varicella-zoster virus (VZV) immunoglobulin G (IgG)	1.94 index value	> 1.1 positive
VZV IgM	0.52 index value	> 1.1 positive
Herpes simplex virus (HSV) 1+2 IgG	0.51 index value	>1.1 positive
HSV 1+2 IgM	0.52 index value	>1.1 positive

The patient was initiated on penicillin G intravenously, four million units four hourly, to be continued for 10 days. By day one, the vision had improved symptomatically, and an OCT showed an early cystic space in the inner retinal layers. By day eight, the visual acuity had improved to 6/7.5 in the left eye, with a significant resolution of the macular chorioretinitis. A repeat OCT in the affected area revealed multiple cystic edematous spaces within the inner retinal layers (Figure 1C). A diagnosis of immune-mediated CME consistent with JHR was made, and he was started on oral prednisolone (40 mg daily, in tapering doses). By day five of steroid treatment, there was a reduction in the cystic spaces, and a subsequent OCT scan on day 42 of follow-up (Figure 1D) confirmed a further decrease in the cystic spaces with a reduction in the central subfoveal thickness from 306 to 270 microns.

## Discussion

Ocular manifestations of syphilis may be seen in 0.6%-2% of all patients, and a wide spectrum of ocular involvement exists that includes scleritis, intermediate uveitis, retinitis, papillitis, and acute posterior placoid chorioretinitis [1]. Zhu et al. studied 41 eyes of 28 HIV-negative patients and described uveitis, vasculitis, chorioretinitis, and optic nerve disease in their cohort [2]. Hughes et al. described 12 patients and noted the presence of peripheral retinitis as the commonest clinical finding (seen in seven), especially amongst the HIV-positive group; HIV-negative patients often present with vitritis, multifocal choroiditis, scleritis, and papillitis [3].

The JHR is a transient immunological reaction usually seen within 24 hours of initiating antimicrobial treatment and presents with symptoms such as fever, chills, or myalgias. The JHR is thought to occur due to lipoprotein release, which induces an inflammatory state. Of these lipoproteins, Tp47 is highly immunogenic and leads to the release of pro-inflammatory cytokines, including tumor necrosis factor-alpha (TNF-a), interleukin (IL)-6, and IL-8 [4].

The ocular correlates of JHR are rare. Fathilah et al. reported the case of a 22-year-old female patient with a positive TPHA test who presented with bilateral retinal vasculitis. Six hours after the initiation of penicillin, she developed a JHR suggested by fever, chills, and headache. Simultaneously, she noticed a reduction of vision in her right eye and was found to have an optic disc and macular edema. She responded to 60 mg of oral prednisolone daily [5]. Abd Manan et al. described the clinical features of a 31-year-old seropositive for HIV infection and syphilis who presented with bilateral disc edema following oral doxycycline treatment for left eye neuroretinitis. He responded fully to oral corticosteroids [6]. Ramtohul et al. described the findings of a 43-year-old man with Lemierre syndrome (internal jugular vein thrombosis following acute ear and throat infections, due to *Fusobacterium necrophorum*), who developed bilateral blurred vision following antibiotic treatment. Fundus evaluation revealed macular edema and peripheral retinal vasculitis bilaterally. An intravitreal injection of ranibizumab 0.5 mg (0.05 mL) was effective in resolving the inflammation [7].

The patient we described had systemic syphilis with latent tuberculosis. The multiple normal OCT scans done before treatment suggest that the cystoid macular edema that developed post penicillin treatment is consistent with a diagnosis of JHR-induced CME. This could potentially be a cause of visual morbidity even in systemically successfully treated cases. Awareness should prompt ophthalmologists to advise regular OCT scans to detect and treat JHR-induced CME following the initiation of treatment.

## Conclusions

Syphilis is a common infectious disease that can cause a range of ocular lesions, including intermediate uveitis, retinitis, and acute posterior placoid chorioretinitis. The appropriate systemic antibiotic treatment may incite an immunological reaction, the JHR, that commonly presents with systemic symptoms or ocular lesions in a small subset of patients. The patient we describe developed CME as a manifestation of JHR following treatment for syphilitic chorioretinitis, which was successfully treated with systemic steroids. Physicians should be aware of the possibility of developing ocular JHR, especially macular edema, and the potential for visual loss.
